# Validation of the Arabic version of the Social Communication Questionnaire

**DOI:** 10.1177/1362361318816065

**Published:** 2019-01-03

**Authors:** Mohammed Aldosari, Eric Fombonne, Hesham Aldhalaan, Mohammed Ouda, Saba Elhag, Hawraa Alshammari, Iman Ghazal, Asma Alsaleh, Tala Alqadoumi, Richard Thomson, Mohanad Al Khasawneh, Mohamed Tolefat, Fouad Alshaban

**Affiliations:** 1 Cleveland Clinic Foundation, USA; 2 Oregon Health & Science University, USA; 3 Center for Autism Research, Saudi Arabia; 4 National Guard Health Affairs, Saudi Arabia; 5 Neurological Disorders Research Center, Qatar Biomedical Research Institute, Hamad Bin Khalifa University, Qatar; 6 King Saud University, Saudi Arabia; 7 Qatar University, Qatar; 8 Shafallah Center for Children with Disabilities, Qatar

**Keywords:** Arabic, autism spectrum disorder, cutoff values, early intervention, epidemiology, screening, Social Communication Questionnaire, validity

## Abstract

Validated screening and diagnostic tools for autism spectrum disorder for use in Arabic-speaking individuals are scarce. This study validated the Arabic version of the Social Communication Questionnaire. The total study sample included 206 children with autism spectrum disorder and 206 typically developing children (73.8% male; mean age: 8.5 (standard deviation = 2.6) years). The mean Social Communication Questionnaire total score was significantly higher in autism spectrum disorder children than in typically developing children (*p* < 0.0001). Scores on the three Social Communication Questionnaire subscales also differed significantly between the groups (*p* < 0.001). Of the 39 items, 37 were endorsed significantly more often in the autism spectrum disorder group. The total Social Communication Questionnaire score did not vary by age or gender. Internal consistency was excellent (alpha = 0.92). In the receiver operating characteristic analysis, the area under the curve for the total score showed excellent discrimination between autism spectrum disorder and typically developing children (area under the curve = 0.95; 95% confidence interval: 0.93–0.97). The areas under the curve for the scale subscores were 0.923 (95% confidence interval: 0.898–0.949) for the social interaction score, 0.872 (95% confidence interval: 0.838–0.905) for the communication score, and 0.856 (95% confidence interval: 0.819–0.893) for the repetitive behaviors score. The findings support the use of the Arabic Social Communication Questionnaire to successfully differentiate children with clinically diagnosed autism spectrum disorder using the established cutoff value for the English version.

## Introduction

Autism spectrum disorder (ASD) is a neurodevelopmental disorder that is characterized by impairments in communication and social interaction, repetitive behaviors, and limited areas of interest that manifest in the first 3 years of life (American Psychiatric Association (APA), 2013). Early diagnosis and intervention have been shown to significantly improve cognitive and adaptive behavior and reduce the severity of ASD (Dawson et al., 2010). In addition, early clinical intervention remarkably decreases the financial burden of ASD, and the estimated cost savings have been shown to outweigh the costs of early intensive behavioral intervention programs (Peters-Scheffer et al., 2012). Research has shown that early detection and ensuing intervention can be achieved with the use of validated screening tools (Allen et al., 2007). However, limited access to cross-culturally validated screening and diagnostic tools for ASD poses major challenges to clinicians and researchers worldwide. Moreover, there are few validated screening tools for Arabic-speaking individuals (Mohamed et al., 2016; Seif Eldin et al., 2008).

Before initiating a country-wide autism epidemiological study, we needed to translate a screening tool and evaluate the overall screening properties of that tool within the local population. We also wanted to determine which cutoff values were associated with optimal values for specificity and sensitivity in order to conduct a large, population-based epidemiological study of ASD.

## Materials and methods

### Participants

Children were recruited from two neighboring Arabian Gulf countries, Qatar and Saudi Arabia. Expatriate residents were excluded when their primary language was not Arabic. The total study sample comprised 412 children: 206 children with an established clinical diagnosis of ASD and 206 gender-matched typically developing (TD) children attending mainstream primary schools.

The sample with ASD was recruited from 10 autism centers and special education schools in Saudi Arabia (*N* = 93) and from the main autism center in Qatar (Shafallah Center for Children with Special Needs; *N* = 113). To be included in the ASD sample, children had to be aged between 5 and 12 years and have a clinically confirmed diagnosis of ASD obtained through a multidisciplinary evaluation. All subjects met the full criteria for ASD as defined by the *Diagnostic and Statistical Manual of Mental Disorders* (5th ed.; DSM-5; APA, 2013). Diagnosis was established by experienced clinicians by combining all developmental history, clinical observations, and examinations and using established diagnostic tools, including the Childhood Autism Rating Scale, the Autism Diagnostic Interview—Revised (ADI-R), and the Autism Diagnostic Observation Schedule. Children with incomplete clinical information were excluded. The ASD sample was recruited by distributing the Social Communication Questionnaire (SCQ—Lifetime version) forms to the centers directly by the study’s researchers.

The TD group was recruited from a total of 20 primary schools in Qatar (*N* = 120) and Saudi Arabia (*N* = 86). To be included, children were aged 5–12 years, were enrolled in regular classrooms, and had no developmental, behavioral, or academic concerns by parental and teacher report. We excluded children with learning disabilities. TD children were matched by gender with the ASD children on a 1:1 ratio.

In Qatar, the comparison sample was recruited by mailing the SCQ forms to the families. The Saudi sample was recruited by distributing forms to the schools. In both instances, the SCQ lifetime version was sent alongside instructions for the parents to complete it. No individual data were collected on caregiver socioeconomic status (SES) or education level but recruitment sites provide services to a diverse population in each country.

### Data collection tools

#### SCQ

The SCQ is a parent-report questionnaire that evaluates three major aspects of ASDs: communication, social interaction, and repetitive behaviors. The SCQ aids in identifying patients who require further evaluation for ASD. The development of the SCQ was modeled after the Autism Diagnostic Interview to generate a brief, parent-completed, screening tool (Berument et al., 1999). The questionnaire exists in two forms: lifetime and current. The “lifetime” form evaluates the patient’s developmental history as well as current behaviors, whereas the “current” form assesses the child’s behavior during the past 3-month period only. It is conveniently brief and relatively inexpensive with 40 questions per form with “yes” or “no” responses that can be answered in less than 10 min. Each item is scored as 0 or 1, and the sum of 39 items yields a total SCQ score ranging from 0 to 39. (Question no. 1 documents whether or not the child has phrase speech and does not have any scoring value.) On the basis of the original validation study based on a clinical sample, cutoffs of 15 and 22 have been proposed to select children likely to have a broader or narrower form of ASD (Berument et al., 1999). In subsequent epidemiologic studies, a cutoff of 12 has been proposed to optimize SCQ performance in population-based samples (Eaves et al., 2006).

#### Arabic version of the SCQ

For the purpose of this study, the English version of the SCQ was translated into Arabic by the study authors (M.S.A. and F.A.A.). Following the initial translation, each item was reviewed and culturally adapted to minimize barriers to comprehension and improve rates of completion by the study participants. One of the few changes made to the original instrument was to remove references to the British rhymes “The Mulberry Bush” and “London Bridge is Falling Down” as the examples used for item 34. The two authors worked with the publisher (Western Psychological Services) to have an SCQ author-assigned reviewer revise the back-translation. Following multiple revision cycles, the final version of the Arabic SCQ was approved by the study’s authors and the publisher.

### Statistical methods

SCQ data were entered into an Excel (Microsoft) spreadsheet as raw scores. The statistical analysis was done in SPSS (IBM Corp.) by one of the authors (E.F.). Conventional statistical tests (Student’s *t*-tests, analyses of variance (ANOVAs), chi-square test, and Fisher’s exact test) were performed to compare continuous and categorical variables. Internal consistency was measured with Cronbach’s alpha coefficient. Receiver operating characteristic (ROC) analysis was performed to examine the overall performance of the SCQ and to estimate sensitivity and specificity for different cut points (Fombonne, 1991). Throughout, 0.05 was retained as the level for statistical significance.

### Ethical approval

The research design and methods were approved by both the Qatar Biomedical Research Institute and the Cleveland Clinic institutional review boards.

## Results

### Participants

Sample characteristics of the study participants are summarized in Table 1. The study sample included 412 children (206 ASD and 206 TD). The majority of participants (56.6%) were from Qatar. There was an overall boy-to-girl ratio of 2.8:1 (73.8% male). Male over-representation was similar in the groups because of matching on gender when selecting the two groups. The mean age of the sample was 8.46 (standard deviation (SD) = 2.65) years, with no significant difference between the ASD group and the comparison group when age was treated either as a continuous or as a categorical variable (see Table 1).

**Table 1.** Sample characteristics (*n* = 412).

	ASD children (*n* = 206)	TD children (*n* = 206)	*p*-Values^a^
Mean age, years (SD)	8.43 (2.6)	8.5 (2.7)	NS
Age group, years (*n*)			
5–6	66	63	
7–8	40	43	
9–10	59	57	NS
⩾11	41	43	
Site (*n*)			
Qatar	113	120	
Saudi Arabia	93	86	NS
Gender male, *n* (%)	152 (73.8)	152 (73.8)	NS
SCQ score, mean (SD)			
Total score	20.2 (6.7)	6.4 (4.1)	<0.0001
Social interaction subscore	8.4 (3.9)	1.9 (2.0)	<0.0001
Communication subscore	6.5 (2.2)	3.0 (1.9)	<0.0001
Repetitive behaviors subscore	4.1 (2.0)	1.2 (1.7)	<0.0001

ASD: autism spectrum disorder; TD: typically developing; SD: standard deviation; SCQ: Social Communication Questionnaire.

a Chi-square test or Fisher’s exact test for categorical variables; *t*-tests for continuous variables.

### SCQ scores in ASD and TD children

**Figure 1.**
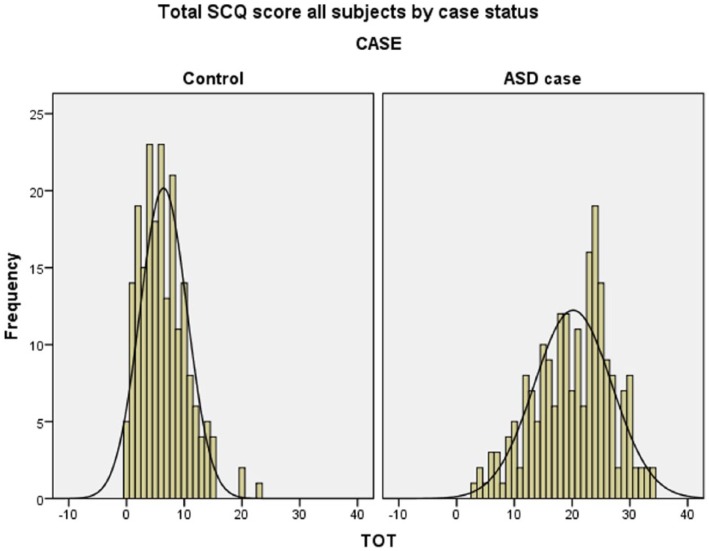
Total score on the Social Communication Questionnaire (SCQ) in the children with autism spectrum disorder (ASD) and the typically developing comparison group.

The distribution of total SCQ scores in the ASD and TD groups is shown in Figure 1. As expected, variability was somewhat larger in the children with ASD than in the TD children as illustrated by the SDs. The difference between total SCQ scores was highly significant (*p* < 0.001), with a mean difference of 13.8 points between the two groups (Table 1). The three subscales of SCQ scores also differed significantly between the two groups (all *p*-values < 0.0001). The corresponding effect sizes for these differences as measured by Cohen’s d were all very large: for the SCQ total score, 2.5; for the social interaction score, 2.1; for the communication score, 1.6; and for the repetitive behaviors score, 1.5.

### Age and gender effects

Separate ANOVAs were performed on the four SCQ scores as dependent variables with gender and age as two- and four-level independent factors. For the total SCQ score, there was no effect of gender (*p* = 0.18) or age group (*p* = 0.70) and no significant interaction between gender and age group (*p* = 0.67). For the social interaction subscore, there was no significant effect of gender (*p* = 0.59) or age group (*p* = 0.95) or their interaction (*p* = 0.87). For the communication score, there was no interaction effect (*p* = 0.10) and no significant effect of age group (*p* = 0.09); however, gender was significant (*p* = 0.008), with girls showing fewer impairments than boys (4.13 vs 4.96). The difference was more pronounced for girls aged 7–8 years and 9–10 years. Finally, for the repetitive behaviors score, no significant effect for gender (*p* = 0.21), age group (*p* = 0.70), or their interaction (*p* = 0.90) was detected.

### Internal consistency

We measured the reliability of the SCQ using Cronbach’s alpha coefficient. All measures of internal consistency were high to satisfactory. For the total SCQ score, alpha was 0.916 when we used all 39 items of the SCQ (after exclusion of item 1). Because 6 items (items 2–7) are not applicable to nonverbal subjects, we re-estimated the reliability coefficient for the 33 items (items 8–40) that applied to all subjects. The corresponding value for alpha was 0.929. Internal consistency estimates were as follows for the three SCQ subscales: 0.901 for the social interaction subscale (15 items; *n* = 401), 0.708 for the communication subscale (13 items; *n* = 293), and 0.818 for the repetitive behaviors subscale (8 items; *n* = 290). Fewer subjects were available for the latter two analyses as values were missing for nonverbal subjects for six items (one in the repetitive behaviors subscales (item 7) and five in the communication subscale (items 2–6)).

### Item discriminant ability

We evaluated the discriminant ability of each item by comparing their frequency in ASD and TD children (Table 2). We calculated odds ratios (ORs) to estimate the magnitude of the association with case-control status. Of 39 comparisons, all but 2 were significant, indicating that 37 of 39 items of the SCQ had significantly higher frequencies among children with ASD than in the comparison group. The two items that did not discriminate between the two groups were item 4 (communication, ever coding; inappropriate questions or statements; OR = 1.3: NS) and item 13 (repetitive behaviors, ever coding; unusually intense special interests; OR = 1.2: NS). For the remaining 37 items, OR point estimates ranged from a low of 0.6 (item 23; communication, age: 4–5 years; use of gestures) to a high of 83.5 (item 40; social interaction, age: 4–5 years; group or cooperative play with peers). Abnormal scores for item 23 were endorsed significantly more often by TD children than by children with ASD (56.6% vs 45.6%, respectively; *χ*^2^ = 4.9, *df* = 1; *p* < 0.05).

**Table 2.** Discriminant validity of the SCQ items (*n* = 412).

Item no.	Period	Item label	Domain	OR	*p*-Values, chi-square	Frequency (%)	
						TD Children	ASD Children
Items 1–7 only applicable to verbal children^a^							
2^b^	Current	Conversation	C	23.2	82.9***	3.7	47.3
3^b^	Ever	Stereotyped utterances	C	8.3	64.3***	30.2	78.2
4^b^	Ever	Inappropriate questions	C	1.3	0.55 NS	14.2	17.4
5^b^	Ever	Pronoun reversal	C	6.4	51.9***	18.4	59.1
6^b^	Ever	Neologisms	C	2.4	11.22***	22.6	40.9
7^b^	Ever	Verbal rituals	R	11.5	80.2*** *	12.8	63.0
Items 8–40 applicable to all children							
8	Ever	Compulsions and rituals	R	7.3	79.8*** *	12.7	51.7
9	Ever	Facial expression	S	7.1	49.6***	7.3	35.9
10	Ever	Use of other’s body to communicate	S	15.4	143.1***	16.5	75.2
11	Ever	Unusual preoccupations	R	5.3	39.1***	9.2	34.8
12	Ever	Repetitive use of objects	R	6.7	73.2***	17.0	57.8
13	Ever	Circumscribed interests	R	1.2	0.7 NS	29.1	33.0
14	Ever	Unusual sensory interests	R	7.5	83.2***	17.5	61.5
15	Ever	Hand and finger mannerisms	R	30.1	167***	6.8	68.8
16	Ever	Complex body mannerisms	R	8.8	97.4***	19.0	67.3
17	Ever	Self-injury		9.2	57.4***	5.9	36.5
18	Ever	Attached to objects		6.5	59.6***	11.7	46.1
19	Current	Friends	S	7.3	85.2***	21.4	66.5
All items are for age 4–5 years							
20	Age 4–5	Social chat	C	19.4	162.4***	19.6	82.5
21	Age 4–5	Imitation	C	5.9	66.0***	18.4	57.3
22	Age 4–5	Pointing to express interest	C	2.5	21.3***	42.4	65.2
23	Age 4–5	Gestures	C	0.6	4.9*	56.6	45.6
24	Age 4–5	Nodding to mean yes	C	7.0	83.3***	28.6	73.7
25	Age 4–5	Head shaking to mean no	C	3.9	43.7***	31.6	64.1
26	Age 4–5	Eye gaze	S	8.0	70.1***	10.2	47.6
27	Age 4–5	Social smiling	S	13.1	73.2***	4.9	40.3
28	Age 4–5	Showing and directing attention	S	6.3	59.8***	12.6	47.6
29	Age 4–5	Offering to share	S	12.3	116.1***	13.2	65.0
30	Age 4–5	Seeking to share enjoyment	S	14.6	113.4***	8.7	58.3
31	Age 4–5	Offering comfort	S	14.2	115.8***	9.8	60.5
32	Age 4–5	Quality of social overtures	S	1.8	6.6**	20.9	32.0
33	Age 4–5	Range of facial expression	S	18.5	94.2***	4.4	45.9
34	Age 4–5	Spontaneously join in social games	C	10.2	79.8***	8.3	47.8
35	Age 4–5	Pretend or make-believe games	C	17.8	156.6***	18.4	80.1
36	Age 4–5	Interest in children	S	5.0	60.0***	28.4	66.5
37	Age 4–5	Response to other children’s approaches	S	9.7	82.8***	9.7	51.0
38	Age 4–5	Look up and pay attention		13.4	102.6***	8.3	54.6
39	Age 4–5	Imaginative play with peers	S	23.1	176.9***	17.5	83.0
40	Age 4–5	Group play	S	83.5	171.8***	2.0	62.4

SCQ: Social Communication Questionnaire; OR: odds ratio; TD: typically developing; ASD: autism spectrum disorder; C: communication subscale; R: repetitive behaviors subscale; S: social interaction subscale.

a Item no. 1 documents whether or not the child has phrase speech and does not have scoring value.

b Analyses for items 2–7 were based on fewer subjects (*n* ranging from 295 to 300) due to items being skipped by parents because of lack of sufficient language.

* *p* < 0.05; **p < 0.01; ***p < 001.

For the 13 items on the communication subscale (1 for the current period, 4 for the ever period, and 8 for the age 4–5 years period), ORs ranged from 0.6 (item 23; use of gestures; age: 4–5 years) to 23.2 (item 2; current period; able to have to and fro “conversation”), with a median OR of 6.4 (item 5; ever period; pronouns mixed up). For the 15 items on the social interaction subscale (1 current, 2 ever, and 12 age 4–5 years), ORs ranged from 1.8 (item 32; coordinated requesting; age: 4–5 years) to 83.5 (item 40; age: 4–5 years; group play cooperative with peers), with a median OR of 12.3 (item 29; age: 4–5 years; offering to share). For the 8 items on the repetitive behaviors subscale (all ever codings), the ORs ranged from 1.2 (item 13; unusually intense special interests) to 30.1 (item 15; motor mannerisms), with a median value of 7.4. Thus, on average, social interaction items had a stronger association with case status than did items in the other two domains.

### Correlations between SCQ scores

We computed the total score in two ways. First, we computed a nonverbal total by summing the scores for items 8–40 (excluding the six items (2–7) that require sufficient language). Next, we calculated the total as previously for nonverbal subjects but as the sum of items 2–40 for verbal subjects. Pearson’s correlation between these two scores was 0.983 (*p* < 0.01) in the overall sample and 0.961 (*p* < 0.01) among ASD children only, suggesting that no imputation technique to adjust for unequal number of items between verbal and nonverbal total scores was necessary. In the whole sample, there was a strong correlation between social and communication scores (Pearson’s *r*: 0.731; *p* ⩽ 0.001) and slightly lower correlation between the repetitive behaviors score with either the social score (Pearson’s *r*: 0.522; *p* ⩽ 0.001) or the communication score (Pearson’s *r*: 0.521; *p* < 0.01). When analyses were repeated in the ASD subsample (*n* = 206), the same pattern emerged, although associations were in general weaker, especially with the repetitive behaviors score. The corresponding coefficients were as follows: for the social and the communication scores (*r* = 0.593; *p* < 0.01), for the repetitive behaviors and the social scores (*r* = 0.178; *p* = 0.01), and for the repetitive behaviors and communication scores (*r* = 0.293; *p* < 0.01). No significant correlation was found with age. All results were the same when nonparametric Spearman’s *r* coefficients were calculated (data not shown).

### Discriminant validity of SCQ scores in ROC analyses and cutoff performances

**Figure 2.**
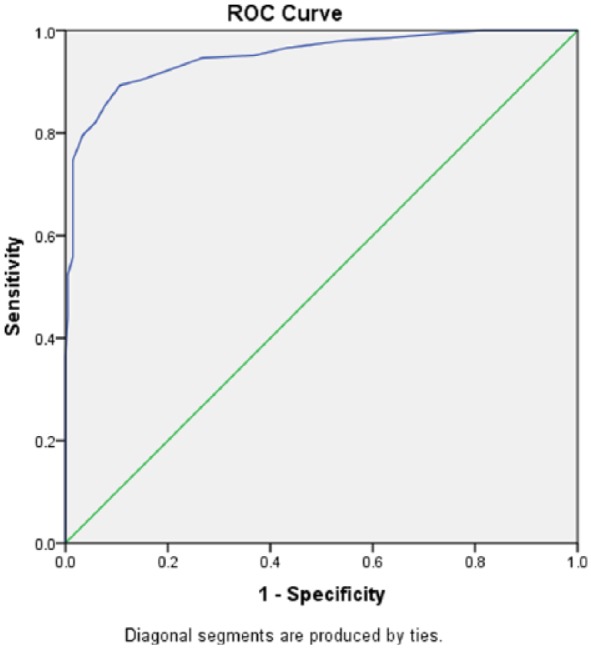
Discriminant validity of the Social Communication Questionnaire (SCQ) total score in the receiver operating characteristic (ROC) curve analysis (*n* = 412).

We used ROC analysis to summarize the overall discriminant validity of the SCQ. For the total score, the area under the curve (AUC) was 0.95 (95% confidence interval (CI): 0.93–0.97) (see Figure 2). For the subscale scores, the AUCs were 0.923 (95% CI: 0.898–0.949) for the social interaction score, 0.872 (95% CI: 0.838–0.905) for the communication score, and 0.856 (95% CI: 0.819–0.893) for the repetitive behaviors score.

For the established cutoff of 15, sensitivity and specificity were 0.796 and 0.966, respectively. For a cutoff of 12, the values for sensitivity and specificity were 0.893 and 0.893, respectively. In-between values for the cutoff were associated with similar overall performance as defined by the sum of the sensitivity and specificity-1 (Youden’s index). Thus, for cutoffs ranging from 11 to 15, the sensitivity varied between 90.3% and 79.6%, whereas the specificity varied between 85.4% and 96.6%. Within that range, Youden’s index had a narrow range of variation between 75.7% and 78.6%.

## Discussion

Our study is the first to assess the use of the SCQ screening tool in an Arabic-speaking population, which worldwide is estimated to number 420 million individuals (United Nations Department of Economic and Social Affairs, 2017). The need to validate a screening tool in Arabic was recognized by the authors while planning a country-wide autism epidemiological study for the Qatari elementary school population. The Modified Checklist for Autism in Toddlers (M-CHAT) is the only available validated screening tool for Arabic populations (Mohamed et al., 2016; Seif Eldin et al., 2008). However, the M-CHAT is restricted to a young and narrow age group, which made it inadequate for our planned study. We initially translated and piloted the Social Responsiveness Scale (SRS), which is among the most widely used quantitative parent/teacher report tools for use in general population, educational, and clinical settings (Bölte et al., 2008; Constantino and Gruber, 2005). After analyzing the pilot data, however, we noted a large proportion of partially completed forms, a fact that many parents attributed to the “long” time needed to complete the scale and the multiple, at times confusing, response formats for each item. We then switched to the SCQ, previously known as the Autism Screening Questionnaire (Berument et al., 1999; Rutter et al., 2003), because it required less time to complete and the response to each item was binary: yes or no. Moreover, and contrary to the SRS that focuses on current behavior only, the SCQ screens the lifespan of the individual, thus allowing highly suggestive ASD features to be included in the screening score even though they may reflect past, but no longer current, behaviors. As such, the SCQ better approximates the longitudinal developmental perspective recommended for autism in diagnostic schemes such as the *DSM* and the *International Classification of Diseases* and that is also embodied in diagnostic tools such as the ADI-R.

The strength of our study resides in the large sample size and the inclusion of subjects from two Arabic-speaking countries, Qatar and Saudi Arabia, which have many similarities in their demographic and ethnic characteristics. These countries also have in common high consanguinity rates among their populations in the order of 40% to 60% (Tadmouri et al., 2009). Qatar’s population is estimated to be around 2.6 million and the Kingdom of Saudi Arabia 32 million. Conducting population research on ASD, especially in this region of the world, can be challenging due to the potential stigma associated with ASD and other developmental conditions and limited research infrastructure. Another feature of our study is the translation process, which was performed in collaboration with an author-assigned reviewer with multiple rounds of translation and back-translation to ensure linguistic equivalence and cultural appropriateness while maintaining the screening performance of the original English version.

We included children between the ages of 5 and 12 years because this is the age group in which autism diagnosis is most reliable (Charman et al., 2005). This age group is also similar to that targeted by our large epidemiological study as well as other major population studies such the Korean prevalence study (Kim et al., 2011), the Mexico study (Fombonne et al., 2016), and the studies of the Centers for Disease Control and Prevention (Baio et al., 2018). Controls were matched by age and sex to enable a better comparison between the two groups. The SCQ showed a high internal consistency coefficient as measured by Cronbach’s alpha coefficient for the total as well as the subscores, which was comparable to other SCQ validation studies (Avcil et al., 2015).

As expected, SCQ scores and subscores were significantly different between the two groups. The corresponding effect sizes for these differences were very large, more so for the total SCQ score, although the subscores also showed highly significant differences. Notably, the social interaction scores and items appeared to have the best discriminant properties compared to the other two domains. Thus, the social scale subscore had the largest overall discriminant validity as measured by the effect size and the AUC from the ROC curves, and it had the highest reliability. In examining item-level discriminant ability, social interaction items showed the more robust associations with case-control status compared with items from the other two domains, a result that is in line with social symptoms being at the core of autism impairment.

When inspecting performances associated with different cutoffs, the sensitivity and specificity were 0.796 and 0.966 for the cutoff of 15. The original SCQ validation study showed sensitivity of 0.86 and specificity of 0.78 for the same cutoff (Berument et al., 1999). Few translated versions showed comparably high sensitivity and specificity, including the Turkish version (sensitivity = 0.94 and specificity = 0.84) and the Mandarin Chinese version (sensitivity = 0.957 and specificity = 0.825) (Avcil et al., 2015; Guo et al., 2011). Age and gender effects within this primary school age range were absent or minimal, a result that supports the use of a single threshold to screen boys and girls within that age range. Thus, our study shows that, for the established cutoff of 15, the sensitivity and specificity of the Arabic SCQ was comparable to figures from previous studies and supports the use of the Arabic SCQ questionnaire as a screening instrument for epidemiological purposes in primary school age samples. In addition, we also determined that the SCQ total score achieved slightly better discrimination between children with ASD and a comparison group than did SCQ subscores, supporting use of the whole scale rather than a shorter version, especially because the full SCQ is well accepted and can be rapidly completed by caregivers.

One limitation of our study is that, due to our sampling procedures, children with ASD had mostly moderate-to-severe impairments, whereas control children were without learning or behavioral problems. It is therefore possible that the strong discriminant ability obtained with the SCQ to differentiate our two samples may have been slightly overestimated. If so, use of the SCQ in our population-based study to screen more representative samples of both cases and controls might show a lower performance. However, the high levels of specificity and sensitivity obtained in this preliminary study were robust and should help maintain good psychometric properties in a different sampling context. A second limitation is that samples were recruited for convenience rather than for being representative of each site or recruitment source, and thus, results might be sensitive to some undocumented selection biases. However, the large sample size and the recruitment across multiple sources and sites should have protected our study against substantial biases and atypical findings. Finally, absence of ASD among controls was evaluated by parental and teacher reports only. However, in the unlikely eventuality that some TD children might have had ASD, it would have contributed to decrease SCQ discriminant accuracy rather than the other way around.

## Conclusion

Our study suggests that the Arabic version of the SCQ can differentiate children with clinically diagnosed ASD from TD children by use of the established cutoff value of 15. Therefore, the Arabic version of the SCQ will be useful in clinical settings for screening children suspected to have autism as well as for executing epidemiologic studies.

## Supplemental Material

AUT816065_Lay_Abstract – Supplemental material for Validation of the Arabic version of the Social Communication Questionnaire Click here for additional data file. Supplemental material, AUT816065_Lay_Abstract for Validation of the Arabic version of the Social Communication Questionnaire by Mohammed Aldosari, Eric Fombonne, Hesham Aldhalaan, Mohammed Ouda, Saba Elhag, Hawraa Alshammari, Iman Ghazal, Asma Alsaleh, Tala Alqadoumi, Richard Thomson, Mohanad Al Khasawneh, Mohamed Tolefat and Fouad Alshaban in Autism
