# Supplementary material for: Validation of the Arabic version of the Social Communication Questionnaire
Source: Autism. 2019 Jan 3;23(7):1655–62. doi: 10.1177/1362361318816065 (PMC6728746; doi:10.1177/1362361318816065)
Supplement: AUT816065_Lay_Abstract – Supplemental material for Validation of the Arabic version of the Social Communication Questionnaire [file AUT816065_Lay_Abstract.pdf]

Lay Abstract

autism

DOI: 10.1177/1362361318816065

Validation of the Arabic version of the Social Communication Questionnaire

Mohammed Aldosari, Eric Fombonne, Hesham Aldhalaan, Mohammed Ouda, Saba Elhag, Hawraa Alshammari, Iman Ghazal, Asma Alsaleh, Tala Alqadoumi, Richard Thomson, Mohanad Al Khasawneh, Mohamed Tolefat and Fouad Alshaban

The Social Communication Questionnaire (SCQ) is a 40-item yes/no questionnaire, that can be completed by parents or primary caregivers of children suspected to have Autism Spectrum Disorder (ASD). It is used to evaluate the communication and social skills of these children, with children who score above the cut-off of 15 usually being referred for further evaluation (whereby a trained professional will seek to confirm whether they have ASD). The aim of the current study was to validate the Arabic version of the SCQ. To do this, we recruited 412 children: 206 who were typically developing and 206 who had ASD. The typically developing children were recruited from 92 different public schools in Qatar, and the children with ASD were recruited through special needs centers/clinics which provide diagnostic and intervention services for children with ASD. The parents or primary caregivers of these children were asked to complete the Arabic version of the SCQ. We then looked at how good the Arabic version of the SCQ was at correctly identifying which children were typically developing and which children had ASD. The findings supported the use of the Arabic SCQ in successfully differentiating between the two different groups of children. This is important as it validates another evidence-based screening tool for children with ASD (using the same cut-off score as the English version). Furthermore, it reaffirmed the reliability of this screening questionnaire to be used across different cultures.
